# Comparison of two methods for assessing weight gain in Brazilian pregnant women

**DOI:** 10.11606/s1518-8787.2026060007244

**Published:** 2026-06-15

**Authors:** Natalia Posses Carreira, Daiane Leite da Roza, Daniela Saes Sartorelli, Maria Carolina de Lima, Lívia Castro Crivellenti

**Affiliations:** I Universidade de São Paulo. Faculdade de Medicina de Ribeirão Preto. Programa de Pós‐Graduação em Saúde Pública. Ribeirão Preto, SP, Brasil; II Universidade de São Paulo. Faculdade de Saúde Pública. Departamento de Epidemiologia. São Paulo, SP, Brasil; III Universidade de São Paulo. Faculdade de Medicina de Ribeirão Preto. Departamento de Medicina Social. Ribeirão Preto, SP, Brasil

**Keywords:** Reference Standards, Weight Gain, Pregnancy, Overweight

## Abstract

**OBJECTIVE:**

To compare two methods for assessing gestational weight gain (GWG) among overweight adult pregnant women receiving care in Primary Health Care.

**METHODS:**

This is a secondary analysis of a randomized controlled clinical trial conducted between 2018 and 2021 involving 260 overweight pregnant women receiving prenatal care at primary health care centers in the municipality of Ribeirão Preto, São Paulo. Participants were assigned to two groups: intervention (received individualized nutritional counseling— GI) or control (received only standard prenatal nutritional guidance—GC). Prenatal weight gain (PWG) was classified according to the U.S. guidelines of the National Academy of Medicine (NAM) and in accordance with Brazilian growth curve recommendations. To assess agreement between the methods, the kappa coefficient was used, and for the classification of weight gain (adequate, insufficient, or excessive), the criteria adopted by each method were used. Logistic regression models were employed to analyze the association between the groups (IG and GC) and the two methods used in the assessment of PWG.

**RESULTS:**

Moderate agreement was observed between the methods, with a pattern of disagreement indicating possible underestimation of GPG by the NAM guidelines. Pregnant women in the GI had a higher likelihood of insufficient GPG according to the Brazilian method (OR = 2.87; 95%CI 0.88–9.25) and a lower likelihood of excessive weight gain according to the US method (OR = 0.63; 95%CI 0.34–1.17), compared to women in the GC, although neither finding was statistically significant.

**CONCLUSION:**

The Brazilian curves proved to be more appropriate for classifying GPG in overweight Brazilian pregnant women, better reflecting weight monitoring during prenatal care. Their adoption may facilitate the early identification of GPG inadequacies and, consequently, contribute to improving the quality of prenatal care.

## INTRODUCTION

Pregnancy weight gain (PWG) is critical for promoting favorable maternal and child health outcomes^1^. Evidence suggests that inadequate, insufficient, or excessive maternal weight gain is associated with an increased risk of maternal and neonatal complications, including gestational diabetes and hypertension; small-for-gestational-age (SGA) or large-for-gestational-age (LGA) newborns ; preterm birth; and postpartum weight retention, among others^2,3^.

Given the growing prevalence of overweight among women of reproductive age^4^ and consistent evidence regarding the relationship between PGW and maternal-fetal outcomes, the Institute of Medicine (now the National Academy of Medicine—NAM) revised its internationally used guidelines for PGW assessment in 2009, establishing specific recommendations based on pre-pregnancy Body Mass Index (BMI)^5^. Preconception BMI is recognized as one of the main determinants of weight gain during pregnancy. In a global meta-analysis involving over one million pregnant women, it was found that 47% of women had excessive PGW due to pre-pregnancy overweight^6^.

Although widely adopted in different countries, there is insufficient evidence that the NAM guidelines are applicable to pregnant women who are not American^7^, since the recommendations were based on the standard of Caucasian women. Furthermore, the epidemiological, nutritional, and cultural differences observed among countries are not considered. Thus, it is essential to develop specific guidelines tailored to the common characteristics of each population^8^.

Since the 1980s, in Brazil, the Ministry of Health (MS) has incorporated various recommendations into its system for assessing nutritional status (NS) and monitoring acute malnutrition, due to the absence of national guidelines^9^. In 2004, the MS began adopting a combination of the Atalah curves^10^, for assessing maternal NS, and the guidelines of the Institute of Medicine^2^, for GPG based on preconception BMI. Although used for years, these guidelines have several limitations, such as the combination of methodologies based on different international indicators; the lack of validation for the Brazilian population or for low- and middle-income countries^12^; the cross-sectional design; and the outdated nature of the Atalah curves. Furthermore, studies indicate low predictive capacity of these curves for adverse neonatal outcomes, such as: preterm and postterm birth, and low birth weight, in addition to underestimation of excess weight^12,13^.

To improve the monitoring of EN and GPG based on pre-pregnancy BMI, national curves and recommendations were developed adapted to the diversity of the Brazilian population. The new tool, which is simple to apply and has been in use in the public health system since 2022^12,14^, can also be used in countries with a similar profile^13^. Its development was based on data from 21 Brazilian studies by the Brazilian Maternal and Child Nutrition Consortium (BMCNC), with internal validation through cross-referencing with data from the Sistema de Vigilância Alimentar e Nutricional (SISVAN) and external validation through the “Nascer no Brasil” study^12,15^.

A study using data from the national “Nascer no Brasil” cohort ^16^ (n = 6,888) compared Brazilian GPG curves with three international references, evaluating the prediction of PIG and GIG births. The curves by Hutcheon et al. ^17^ were developed in a sample of American women; those of Intergrowth-21st^18^, in healthy women residing in urban areas of eight countries (Brazil, China, India, Italy, Kenya, Oman, the United Kingdom, and the United States); and those of the Lifecycle consortium^19^, based on data from 33 cohorts in Europe, North America, and Oceania.

Compared to the references evaluated in the previously mentioned study, the Brazilian curves demonstrated greater predictive power, with distributions of GPG values and proportions of women classified below and above selected percentiles closer to the expected pattern, considering pre-pregnancy BMI and gestational trimester. In contrast, the Intergrowth-21st^18^ and Lifecycle^19^ charts classified a higher frequency of women below the 50th percentile, while those by Hutcheon et al.^17^ overestimated GPG in women classified as eutrophic, overweight, and obese^16^.

A randomized clinical trial^20^evaluated the effectiveness of nutritional counseling in preventing excessive weight gain in overweight pregnant women. The intervention, based on encouraging the consumption of fresh and minimally processed foods over ultra-processed foods and the regular practice of physical activity, reduced the risk of excessive GWG (OR = 0.56; 95%CI 0.32–0.98; p = 0.04). However, maternal weight gain was not analyzed in accordance with current national recommendations.

Assuming that the NAM guidelines, widely used worldwide, have not been validated for the Brazilian population and may underestimate excessive GWG, especially among overweight pregnant women, the hypothesis of this study is that Brazilian- d curves and recommendations offer greater sensitivity in identifying inadequate GWG patterns. The comparison between the methods is relevant, since overweight pregnant women are at higher risk of excessive weight gain during pregnancy, a condition associated with adverse maternal and neonatal outcomes^21^. Thus, the objective of this study was to compare the methods for assessing GPG proposed by the US guidelines (NAM) and by the Brazilian curves and recommendations in overweight pregnant women.

## METHODS

### Study Design and Population

This is a secondary analysis that utilized data from a randomized controlled clinical trial conducted between 2018 and 2021 involving overweight adult pregnant women receiving prenatal care at seven health units (HUs) in the municipality of Ribeirão Preto, SP, Brazil. The study protocol is described in detail in the publication by Sartorelli et al.^22^ The conduct of the clinical trial was approved by the Research Ethics Committee of the Centro de Saúde Escola da Faculdade de Medicina de Ribeirão Preto (69997717.6.0000.5414) and followed the CONSORT guidelines. The clinical trial was registered in the Brazilian Clinical Trials Registry in July 2018 (RBR-2w9bhc).

Trained nutritionists identified potentially eligible women and invited them to participate in the study during their prenatal visits at the antenatal clinics. Low-risk pregnant women aged 18 years or older, with a pre-pregnancy BMI between 25 kg/m^2^ and 29 kg/m^2^ and a gestational age of no more than 15 weeks and 6 days, were included. Women who reported a history of diabetes or current use of oral hypoglycemic agents, insulin, or weight-loss medication were excluded. All participants who agreed to participate in the study signed the Informed Consent Form and were randomly assigned to the intervention group (IG) or control group (GC).

In the clinical trial, the sample size was calculated based on the proposed primary outcome: the proportion of women with excessive GPG. A minimum significance level of 5% (α = 0.05), a power of 90% (β = 0.10), and a 40% loss to follow-up due to the social isolation imposed by the Covid-19 pandemic were considered, resulting in a sample size of 350 pregnant women.

In the present study, the sample size was based on a non-probabilistic convenience sampling strategy^23^, including all women with data for calculating the cumulative GPG for classification according to Brazilian curves. Furthermore, following the modified intention-to-treat principle, women who participated in at least one nutritional counseling session were included in the GI group^24^. Thus, 260 pregnant women were included (GI = 121; GC = 139) (Figure).

**Figure:**
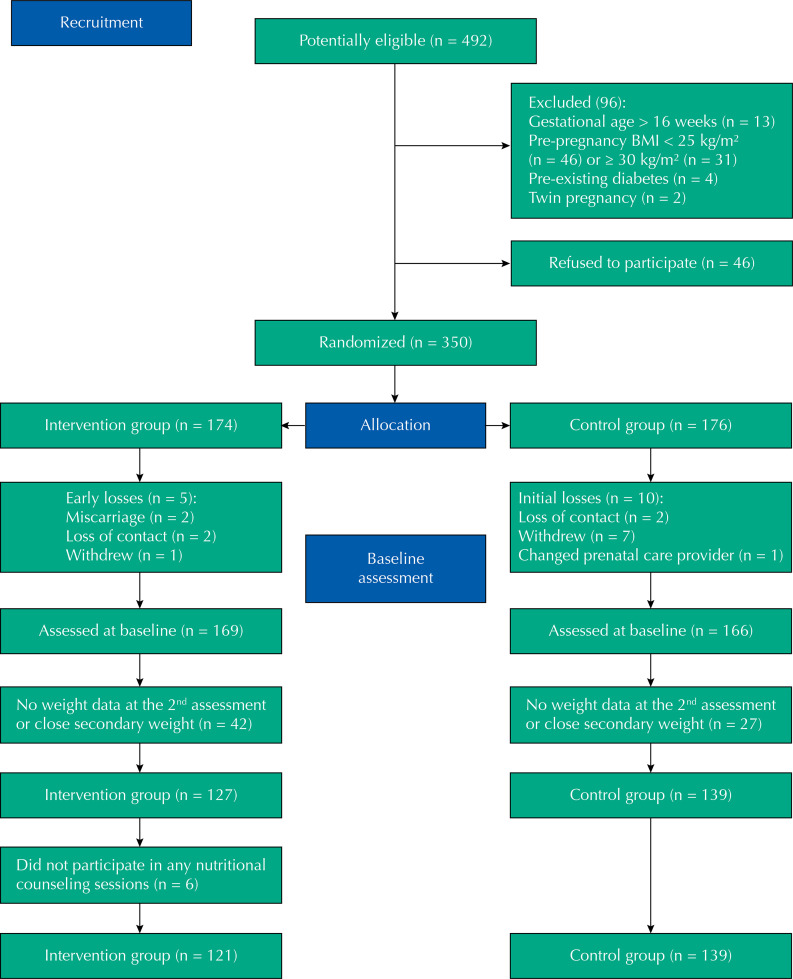
Study flowchart.

### Maternal Characteristics

Sociodemographic, obstetric, and maternal lifestyle data, such as age, marital status, self-reported skin color, paid employment, parity, weekly physical activity, smoking, and alcohol consumption in the past month, were collected by trained nutritionist researchers using a structured questionnaire during the first assessment of the clinical trial.

Participants were randomized using REDCap software^25^, with stratified allocation by antenatal clinic, to ensure balanced distribution between the control and intervention groups. Participants and researchers were aware of the group assignments due to the nature of the nutritional counseling—in-person sessions with trained dietitians—which made blinding impossible.

All participants received standard prenatal care at the HU, including guidance on weight gain, nutrition, and pregnancy-related questions. The intervention group (GI) participated in three individual nutritional counseling sessions, focused on encouraging the consumption of fresh and minimally processed foods over ultra-processed foods, and on regular physical activity. The sessions, conducted by nutritionists, lasted approximately 30 minutes, with an average interval of six weeks.

Pregnant women in the GC received only routine prenatal care at the HU clinic, which includes assessment of weight and height, general guidance on healthy eating, and appropriate weight gain during pregnancy. After delivery, both groups received guidance on returning to their pre-pregnancy weight. Additional details are described in previous publications^20,22^.

### Gestational Weight Gain

Cumulative GPG was calculated based on the difference between the participant’s final weight, obtained on average at 33.5 weeks of gestation, and the estimated pre-pregnancy weight. The participant’s final weight measurement was obtained at the time of the second clinical trial assessment or through access to medical records in the HygiaWeb information system (secondary data). A correlation was performed between the weight values measured at the second study evaluation and the secondary values obtained, yielding a Spearman correlation coefficient of 0.99 (p < 0.01)^20^.

Pre-pregnancy weight was estimated based on the weight measured at the time of clinical trial screening. The weight measured up to the 13th gestational week (GW) was adopted as the pre-pregnancy weight; if measured between the 14th and 15th GW, 0.45 kg and 0.91 kg, respectively, were subtracted from the measured weight^26^. Pre-pregnancy BMI was calculated as the ratio of pre-pregnancy weight to the square of measured height, and was classified according to the World Health Organization’s theoretical reference standards, specifically the 1995^27^ for pregnant women over 19 years of age, and for adolescent pregnant women (n = 17) (≥ 18 years and ≤ 19 years), the BMI classification reference by age^28^.

To estimate the average weekly weight gain, the weight measured at the second evaluation of the clinical trial—conducted preferably between the 34th and 36th weeks of gestation—was subtracted from the weight recorded during the second trimester of pregnancy (≥ 12 weeks of gestation). The difference was divided by the number of weeks between the two time points. In the absence of weight records between 34 and 36 weeks of gestation, the last available weight measurement from a prenatal visit prior to 36 weeks of gestation, as recorded in the pregnant woman’s medical chart (secondary data), was used.

### Methods for Assessing Gestational Weight Gain

#### Brazilian Curves and Recommendations

The GPG curves and recommendations for Brazilian pregnant women were used to assess maternal GPG^13^. Cumulative weight gain was classified by determining the exact percentile for a database of participants, based on information regarding cumulative weight gain, gestational age at the last weight measurement, and pre-pregnancy BMI. The percentile was calculated using Microsoft Excel software^29^.

Since the sample consisted exclusively of women who were overweight before pregnancy, the percentile ranges established by this guideline were adopted, whereby pregnant women with weight gain above the 27th percentile were classified as having excessive weight gain, those between the 18th and 27th percentiles as having adequate weight gain, and women below the 18th percentile as having insufficient weight gain^30^.

#### National Academy of Medicine

According to the NAM proposal, average weekly weight gain was classified as: insufficient, adequate, and excessive, considering the recommended weight gain range for pregnant women who were overweight before conception (BMI between 25 kg/m^2^ and 29.9 kg/m^2^). Thus, pregnant women with an average weekly weight gain of less than 0.23 kg were classified as having insufficient GPG, those with a weekly GPG of more than 0.33 kg as having excessive weight gain, and women who had an average weekly weight gain between 0.23 kg and 0.33 kg were classified as having adequate weekly GPG^2^.

## Statistical Analyses

Maternal characteristics at the study baseline were presented as absolute frequency (n) and relative frequency (%), and Pearson’s chi-square test was used to assess the association between these variables according to treatment groups.

To assess the agreement between the two methods of GPG classification, NAM and the national recommendation curves, the weighted Kappa agreement coefficient^31^ was used. The Kappa agreement coefficient was interpreted as follows: less than 0.20 represents poor agreement; 0.20–0.40 represents fair agreement; 0.41–0.60 represents moderate agreement; 0.61–0.80 represents substantial agreement; greater than 0.80 represents high agreement^32^.

The analyses in this study followed the principles of modified intention-to-treat^24^, in which women in the IG who did not participate in any nutritional counseling session were excluded. This approach was adopted considering that the GPG can be modified by prenatal interventions. Thus, to ensure comparability between the groups, the IG included pregnant women who participated in at least one nutritional counseling session, while the GC was not exposed to the intervention.

Crude logistic regression models were used to compare GPG according to monitoring methods and treatment groups, using the “adequate GPG” category as the reference. To select the set of adjustment variables, a Directed Acyclic Graph (DAG)^33^ was constructed^,^including the exposure variable (treatment group), outcome (GPG), and other maternal variables collected in the study (Supplementary Material)^a^.

In the present study, the DAG indicated the absence of confounding variables in the association between treatment group and gestational weight gain, since this is a randomized controlled clinical trial, which already minimizes such biases. Additional adjustments could overestimate the control group and compromise the results^34^. Analyses were performed using SAS software version 9.4 for Windows (SAS Institute Inc., Cary, NC, USA), and p-values < 0.05 were considered statistically significant.

## RESULTS

Of the 335 pregnant women with baseline data, considering the treatment groups (IG = 169 and GC = 166), 266 had weight data available to calculate cumulative and weekly weight gain (IG = 127 and GC = 139). However, following the modified intention-to-treat principle, six pregnant women in the TI group were excluded from the analyses because they did not participate in any nutritional counseling sessions, resulting in a total of 121 pregnant women in the TI group.

Most pregnant women were 25 years old or younger. In both groups, a higher frequency was observed among pregnant women who engaged in less than 150 minutes of physical activity per week and self-identified as mixed-race. A significant difference was observed between the groups regarding paid work, with a higher proportion of pregnant women not engaged in paid work in the GC. For the other maternal characteristics, no significant differences were observed between the groups (Table 1).

**Table 1:** Maternal characteristics at baseline according to treatment group. Ribeirão Preto (SP), 2021, n = 260.

Maternal characteristics	GI (n = 121) n (%)	GC (n = 139) n (%)	p-value^a^
Age (years)			0.55
≤ 25	47 (42.7)	63 (57.3)	
26–34	53 (48.6)	56 (51.4)	
≥ 35	21 (51.2)	20 (48.8)	
Physical activity (minutes/week)			0.78
< 150	86 (46.0)	101 (54.0)	
≥ 150	35 (48.0)	38 (52.0)	
Marital status			0.42
Married/living with partner	94 (48.0)	102 (52.0)	
Other	27 (42.2)	37 (57.8)	
Self-reported skin color			0.81
White	36 (44.4)	45 (55.6)	
Black	17 (45.9)	20 (54.1)	
Brown	66 (47.8)	72 (52.2)	
Education (years of schooling)^b^			0.94
≤ 8	28 (48.3)	30 (51.7)	
9 to 11	79 (45.9)	93 (54.1)	
≥ 12	14 (48.3)	15 (51.7)	
Paid work			**0.02**
Yes	83 (52.5)	75 (47.5)	
No	38 (37.3)	64 (62.7)	
Parity			0.19
Primiparous	40 (41.2)	57 (58.8)	
Multiparous	81 (49.7)	82 (50.3)	
Smoking			0.41
Never smoked	93 (48.9)	97 (51.1)	
Former smoker	10 (37.0)	17 (63.0)	
Current smoker	18 (41.9)	25 (58.1)	
Alcohol consumption in the past 30 days			0.57
Yes	21 (42.9)	28 (57.1)	
No	100 (47.4)	111 (52.6)	

IG: intervention group; GC: control group.

^a^ Referring to Pearson’s chi-square test.

^b^ Data available for 259 women.

The methods for monitoring GPG, according to treatment groups, showed moderate agreement, as indicated by the kappa coefficients. Among the 139 women in the GC, 116 (83.45%) were classified by both methods into the same GPG category. Regarding the IG, of the 121 women, 97 (80.17%) were classified in the same GPG category by both methods (Table 2).

**Table 2:** Agreement analysis between methods of monitoring gestational weight gain according to treatment groups. Ribeirão Preto (SP), 2021, n = 260.

Brazilian curves (2021)	NAM (2009)			Kappa
	Control group (n = 139)			
	Inadequate (n = 12)	Adequate (n = 25)	Excessive (n = 102)	
Insufficient (n = 12)	**10 (83.3)**	2 (8.0)	0 (0)	
Adequate (n = 12)	2 (16.7)	**7 (28.0)**	3 (2.9)	0.55 (95%CI 0.40–0.70)
Excessive (n = 115)	0 (0)	16 (64.0)	**99 (97.1)**	
	Intervention group (n = 121)			
	Insufficient (n = 17)	Adequate (n = 29)	Excessive (n = 75)	
Insufficient (n = 20)	**16 (94.1)**	4 (13.8)	0 (0)	
Adequate (n = 7)	1 (5.9)	**6 (20.7)**	0 (0)	0.58 (95%CI 0.46–0.72)
Excessive (n = 94)	0 (0)	19 (65.5)	**75 (100)**	

NAM: *National Academy of Medicine*; 95%CI: 95% confidence interval.

In bold: frequencies that match across methods.

Sources: Kac et al.^13^(2021); IOM (2009)^2^.

With points located below the main diagonal (in bold), a possible pattern of underestimation of GPG by the US method was identified, regardless of the treatment group. In this regard, in the GC, two pregnant women (16,7%) were classified as having adequate weight gain by the Brazilian curves method but as having insufficient weight gain by the NAM method. Furthermore, 16 pregnant women (64,0%) with excessive weight gain according to the Brazilian curves were classified as having adequate weight gain by the NAM method. Meanwhile, in the IG, one woman (5,9%) was categorized as having adequate GWG by the national method and insufficient by the NAM method, and 19 women (65,5%) with excessive GWG by the national method were categorized as having adequate GWG by the US method (Table 2).

Table 3 presents the GPG assessment data according to the monitoring methods, considering the treatment groups. It was observed that, according to the Brazilian curve method, women in the GI, compared to the GC, had a higher chance of insufficient GPG (OR = 2.87; 95%CI 0.88–9.25) and, according to the NAM method, a lower chance of excessive GWG (OR = 0.63; 95%CI 0.34–1.17), indicating associations in opposite directions and of different magnitudes, although without statistical significance in both methods.

**Table 3:** Assessment of gestational weight gain according to methods of monitoring gestational weight gain by treatment groups. Ribeirão Preto (SP), 2021, n = 260.

Methods	GC	GI	p-value^a^	OR^b^ (95%CI)
NAM	GPG n (%)			
Insufficient	12 (8.6)	17 (14.0)	0.13	1.22 (0.49–3.04)
Adequate	25 (18.0)	29 (24.0)		1.00 (ref.)
Excessive	102 (73.4)	75 (62.0)		0.63 (0.34–1.17)
Brazilian curves				
Insufficient	12 (8.6)	20 (16.5)	0.12	2.87 (0.88–9.25)
Adequate	12 (8.6)	7 (5.8)		1.00 (ref.)
Excessive	115 (82.7)	94 (77.7)		1.40 (0.53–3.70)

GWG: gestational weight gain; 95%CI: 95% confidence interval; GC: control group; IG: intervention group; NAM: *National Academy of Medicine*; OR: odds ratio; ref.: reference.

^a^ Pearson’s chi-square test.

^b^ Odds ratio calculated using logistic regression models, IG as reference group (effect of intervention compared to control).

## DISCUSSION

This was the first national study to compare the method of monitoring GPG using Brazilian growth curves with the method proposed by the NAM among overweight pregnant women. Moderate agreement between the methods was observed using the kappa coefficient, as well as a tendency for the US method to underestimate GPG. Furthermore, using the Brazilian method, an increased likelihood of insufficient GPG was observed among pregnant women in the GI, while the US method indicated a lower likelihood of excessive gain in this same group, although neither finding was statistically significant. These findings may reinforce the pattern of underestimation associated with the US method compared to Brazilian curves, since the method classified a smaller number of pregnant women with excessive weight gain in the intervention group.

Regarding the moderate agreement observed between the methods, there are no studies in the literature that have explored this issue. However, there are studies that have analyzed the predictive capacity of these methods regarding maternal and fetal health outcomes^7,16,35,36^ and indicate limited or modest predictive performance, especially when used in isolation. Furthermore, they suggest that local or regional charts tend to better represent the pattern of the evaluated population, which may confer greater predictive power compared to international recommendations.

Evidence has highlighted that the recommendations for total GPG and average weekly weight gain proposed by the NAM may not be ideal for non-American pregnant women, particularly in developing countries^2,36^. A review of studies in Latin America compared the accuracy of three proposals for assessing maternal weight gain in identifying the risk of inadequate length and birth weight: (1) the Institute of Medicine (IOM) - now NAM, (2) the Rosso-Mardones (RM) chart, and (3) the modified RM chart, proposed by Atalah et al.^36^

In large samples of pregnant women from Chile and Uruguay, the RM chart was found to be more sensitive in identifying high-risk cases. However, predictive values were similar for the three approaches; in general, they showed low positive predictive value and high negative predictive value for the outcomes analyzed^36^. According to the authors, the main limitation to the use of the IOM’s weight gain recommendations in Latin American women is related to the shorter average height of this population—approximately 20 cm shorter than that observed among North American women—a variable that is part of the BMI calculation^5^ and that can also directly influence birth weight^39^. Similarly, the average height of Brazilian women is significantly lower than that of North American women (160 cm *versus* 176 cm, respectively)^5,40^.

In this sense, the findings of the present study are consistent with previous literature, showing only moderate agreement between the methods and a tendency for the US method to underestimate GPG compared to Brazilian curves. This tendency may lead to the classification of Brazilian pregnant women as having insufficient weight gain when the observed gain is consistent with the national anthropometric and biosocial standard and may also reduce the identification of excessive weight gain, particularly in the third trimester.

In a retrospective cohort study conducted in China^35^, involving 13,366 pregnant women, different GPG standards (Intergrowth-21st, IOM, and a Chinese reference) were compared regarding their ability to identify the risk of Fetal Growth Restriction (FGR) and related adverse outcomes. Among the pregnant women, 46.0% gained weight above the IOM recommendations, while 40.0% and 30.0% gained weight above the Intergrowth-21st and Chinese reference recommendations, respectively. On the other hand, it was observed that pregnant women with weight gain above the recommendations for the Intergrowth-21st and Chinese reference standards had increased risks of FGA of 27.0% and 30.0%, respectively. Meanwhile, pregnant women with excessive weight gain according to IOM recommendations had a 22.0% increased risk of GDM, representing lower predictive power.

Furthermore, the literature debates whether the NAM weight gain recommendations are appropriate for all BMI categories. In Germany, a study of over 650,000 births assessed their validity by comparing the prevalence of preterm birth and low birth weight among women who gained weight within or outside the recommendations, stratified by pre-pregnancy BMI. Underweight and normal-weight women who followed the recommendations had a lower prevalence of adverse outcomes. Among overweight and obese women, however, following the guidelines was associated with increased risk. It was concluded that the validity of the recommendations varies according to preconception BMI, highlighting the need for specific guidelines for each group^38^.

Tsai et al.^41^ assessed the appropriateness of the NAM recommendations for Taiwanese women by analyzing the risk of fetal macrosomia. Normal-weight women who gained > 13 kg had a fourfold higher risk of macrosomia, and overweight women who gained > 11.5 kg had a ninefold higher risk. The authors suggested lower limits: < 11.5 kg for normal-weight women and < 10 kg for overweight women, as these values are lower than those of the NAM, which may underestimate excessive weight gain and increase the risk of adverse neonatal outcomes.

In the present study, although no significant difference in GPG was found between the treatment groups—, regardless of the method used—an increased likelihood of insufficient GPG was observed among pregnant women in the Brazilian-method group, while the American method indicated a lower likelihood of excessive gain in that same group.

In a review of randomized clinical trials involving 11 studies (n = 4,422 pregnant women), it was found that interventions based on diet, exercise, or both increased the risk of insufficient PGW compared to the control group (RR = 1.14; 95%CI 1.02–1.27)^42^. However, in the present study, insufficient PGW may have been overestimated due to the methodology used to estimate pre-pregnancy weight, which was obtained by measurement up to the 13th week of gestation, without considering initial weight gain^26^. Recent evidence indicates that weight measured by the 8th week or self-reported weight may provide more accurate estimates of pre-pregnancy weight^15^. Another plausible explanation is that some women in the intervention group may have been more concerned about nutritional counseling due to maternal and child well-being^43^.

The possible pattern of underestimation of PGW by the NAM method may have contributed to the lower proportion of pregnant women classified as having excessive weight gain and, consequently, to the observation of a protective effect of the intervention, albeit without statistical significance, since excessive PGW was the primary outcome in the intervention study. This can be explained by the more permissive thresholds established by NAM compared to Brazilian recommendations, which are more stringent and classified a higher proportion of pregnant women as having excessive weight gain.

In this context, small reductions in maternal weight gain resulting from the intervention may have been sufficient to reduce the number of cases classified as excessive weight gain according to NAM criteria. However, these reductions were not sufficiently significant to impact the higher proportion of women classified as having excessive weight gain according to the Brazilian curves. Thus, the divergence between the methods may reflect differences in the categorization of gestational weight gain, without necessarily indicating variations in the intervention’s effect on this outcome.

In the original study^20^, the intervention proved effective in reducing the risk of excessive GPG, as assessed by the NAM. In the present study, this association did not reach statistical significance. This may be related to the fact that, in previous analyses, the DAG was not employed, and adjustments were made to the models. In the present study, the use of DAG (Supplementary Material) highlighted the absence of adjustment variables, since this is a randomized controlled clinical trial, which contributes to minimizing bias.

Our findings reveal a divergence between the methods for monitoring excessive weight gain (EWG), not only in terms of agreement but also in the assessment of EWG between treatment groups. Despite moderate agreement, a classification pattern below the main diagonal was observed, indicating that the North American method tends to underestimate excessive weight gain compared to the Brazilian curves. This discrepancy is reflected in associations in opposite directions and of modest magnitude, since the Brazilian method identified a higher likelihood of insufficient GPG in the GI (OR = 2.87), while the NAM method indicated a lower likelihood of excessive GPG (OR = 0.63) in that same group.

Although not statistically significant, this difference is clinically relevant, since the method adopted for monitoring can influence the identification of pregnant women at risk—that is, those with weight gain above the recommended level—preventing timely nutritional interventions and more rigorous monitoring, which could prevent adverse maternal and fetal outcomes. Additionally, these differences may impact nutritional surveillance and compromise public policies aimed at preventing excessive weight gain and its health consequences.

The study has some limitations: it was conducted exclusively with overweight pregnant women and cannot be generalized to women in other pre-pregnancy BMI ranges; the methodology used to estimate pre-pregnancy weight may have led to an insufficient overestimation of GPG; the sample size derives from a clinical trial not originally designed for this outcome, although it includes all participants with available data and uses one of the methods considered in the original sample calculation (NAM); blinding was not possible due to the in-person nature of the intervention ; and modified intention-to-treat was adopted to ensure comparability between groups.

Among the study’s strengths, the comparison between the national method for monitoring GPG—recently incorporated into prenatal care in the country’s public health system—and the previously used US method stands out. Furthermore, comparing methods in a population of overweight pregnant women is crucial in the context of public health, as they comprise the group at highest risk for excessive weight gain during pregnancy^44,45^.

The concordance analyses indicated that the US method tends to underestimate GPG compared to the Brazilian method. This result highlights limitations in the applicability of the NAM guidelines to populations other than the US population. Furthermore, the lack of validation of these guidelines for the Brazilian population may contribute to an inadequate classification of GPG, especially in cases of inadequate weight gain (insufficient or excessive).

Therefore, the Brazilian curves and recommendations represent a more reliable monitoring method that is better suited to the profile of Brazilian pregnant women. Their widespread dissemination among healthcare professionals is essential to ensure appropriate monitoring and management during pregnancy, promoting the well-being and health of both mother and child.

## Supplementary Material

Supplementary Material

## Data Availability

The data supporting the results presented in this study are not publicly available but can be made available upon request to the corresponding author.

## Footnotes

a The supplementary material is available on Figshare: [DOI] 10.6084/m9.figshare.31851868 LINK: https://figshare.com/s/c2f4b96ca5432eb88ee5
