# Supplementary material for: Comparison of two methods for assessing weight gain in Brazilian pregnant women
Source: Rev Saude Publica. 2026 Jun 15;60:e18. doi: 10.11606/s1518-8787.2026060007244 (PMC13271210; doi:10.11606/s1518-8787.2026060007244)
Supplement: Supplementary Material [file 1518-8787-rsp-60-e18-suppl01.pdf]

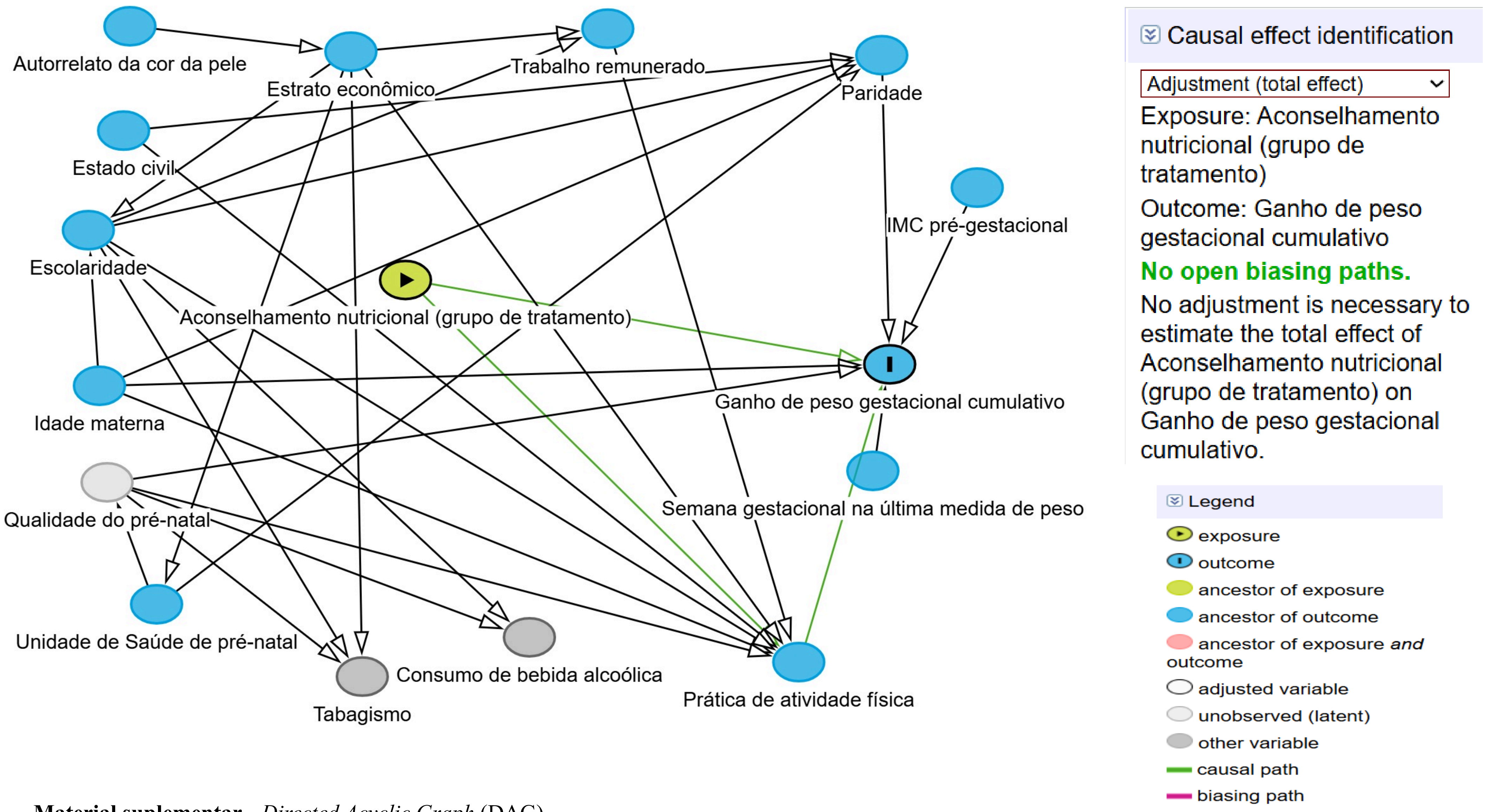

Material suplementar - *Directed Acyclic Graph* (DAG)
