# Supplementary material for: Comparison of two methods for assessing weight gain in Brazilian pregnant women
Source: Rev Saude Publica. 2026 Jun 15;60:e18. doi: 10.11606/s1518-8787.2026060007244 (PMC13271210; doi:10.11606/s1518-8787.2026060007244)
Supplement: Translation

# Translation: Comparação entre dois métodos de avaliação do ganho ponderal em gestantes brasileiras

**Authors:** Natalia Posses Carreira, Daiane Leite da Roza, Daniela Saes Sartorelli, Maria Carolina de Lima, Lívia Castro Crivellenti

**Affiliations:** I Universidade de São Paulo. Faculdade de Medicina de Ribeirão Preto. Programa de Pós‐Graduação em Saúde Pública. Ribeirão Preto, SP, Brasil; II Universidade de São Paulo. Faculdade de Saúde Pública. Departamento de Epidemiologia. São Paulo, SP, Brasil; III Universidade de São Paulo. Faculdade de Medicina de Ribeirão Preto. Departamento de Medicina Social. Ribeirão Preto, SP, Brasil

**Keywords:** Padrões de Referência, Ganho de Peso, Gravidez, Sobrepeso

## Abstract

**OBJETIVO:**

Comparar dois métodos de avaliação do ganho de peso gestacional (GPG) entre gestantes adultas com sobrepeso atendidas na Atenção Primária à Saúde.

**MÉTODOS:**

Trata-se de uma análise secundária de um ensaio clínico randomizado e controlado, realizado entre 2018 e 2021, com 260 gestantes com sobrepeso em acompanhamento pré-natal em unidades básicas de saúde do município de Ribeirão Preto, São Paulo. As participantes foram alocadas em dois grupos: intervenção (receberam aconselhamento nutricional individualizado – GI) ou controle (receberam apenas orientações nutricionais usuais do pré-natal – GC). O GPG foi classificado segundo as diretrizes norte-americanas da *National Academy of Medicine* (NAM) e de acordo com as recomendações das curvas brasileiras. Para avaliar a concordância entre os métodos, empregou-se o coeficiente de concordância de kappa e, para a classificação do ganho ponderal (adequado, insuficiente ou excessivo), utilizaram-se os critérios adotados por cada método. Modelos de regressão logística foram empregados para analisar a associação entre os grupos (GI e GC) e os dois métodos empregados na avaliação do GPG.

**RESULTADOS:**

Observou-se concordância moderada entre os métodos, com um padrão de discordância que indica possível subestimação do GPG pelas diretrizes da NAM. Gestantes do GI apresentaram maior chance de GPG insuficiente pelo método brasileiro (RC = 2,87; IC95% 0,88–9,25) e menor chance de ganho excessivo pelo método norte-americano (RC = 0,63; IC95% 0,34–1,17), em comparação às mulheres do GC, embora ambos sem significância estatística.

**CONCLUSÃO:**

As curvas brasileiras mostraram-se mais apropriadas para a classificação do GPG em gestantes brasileiras com sobrepeso, refletindo melhor o monitoramento do peso durante o pré-natal. Sua adoção pode favorecer a identificação precoce de inadequações no GPG e, consequentemente, contribuir para a qualificação da assistência pré-natal.

## INTRODUÇÃO

O ganho de peso gestacional (GPG) é fundamental para a promoção de desfechos favoráveis à saúde materno-infantil^1^. Evidências sugerem que o ganho de peso materno inadequado, insuficiente ou excessivo, está associado a maior risco de complicações maternas e neonatais, incluindo diabetes e hipertensão gestacional; recém-nascidos pequenos ou grandes para a idade gestacional (PIG ou GIG); parto prematuro; retenção de peso no pós-parto, entre outros^2,3^.

Diante da crescente prevalência de excesso de peso entre mulheres em idade reprodutiva^4^ e de evidências consistentes acerca da relação entre GPG e desfechos materno-fetais, o *Institute of Medicine* (atualmente *National Academy of Medicine* - NAM) revisou, em 2009, suas diretrizes internacionalmente utilizadas para a avaliação do GPG, passando a estabelecer recomendações específicas com base no Índice de Massa Corporal (IMC) pré-gestacional^5^. O IMC pré-concepção é reconhecido como um dos principais determinantes do ganho ponderal durante a gestação. Em uma meta-análise global com mais de um milhão de gestantes, identificou-se que 47% das mulheres apresentaram GPG excessivo, decorrente do excesso de peso pré-gestacional^6^.

Apesar de amplamente adotadas em diferentes países, não há evidências suficientes de que as diretrizes da NAM sejam aplicáveis a gestantes que não sejam norte-americanas^7^, uma vez que as recomendações foram baseadas no padrão de mulheres caucasianas. Além disso, as diferenças epidemiológicas, nutricionais e culturais observadas entre os países não são consideradas. Assim, é fundamental o desenvolvimento de diretrizes específicas direcionadas às características comuns de cada população^8^.

Desde a década de 1980, no Brasil, o Ministério da Saúde (MS) incorporou diferentes recomendações em seu sistema de avaliação do estado nutricional (EN) e monitoramento GPG, devido à ausência de uma diretriz nacional^9^. Em 2004, o MS passou a adotar a combinação das curvas de Atalah^10^, para avaliação do EN materno, e das diretrizes do *Institute of Medicine* ^2^, para o GPG segundo o IMC pré-concepção. Apesar de utilizadas por anos, essas diretrizes apresentaram diversas limitações, como: a combinação de metodologias baseadas em indicadores internacionais distintos; a ausência de validação para a população brasileira ou para países de baixa e média renda^12^; o desenho transversal; e a desatualização das curvas de Atalah. Além disso, estudos apontam baixa capacidade preditiva dessas curvas para desfechos neonatais adversos, como: PIG e GIG e baixo peso ao nascer, além da subestimação do excesso de peso^12,13^.

Para aprimorar o monitoramento do EN e do GPG segundo o IMC pré-gestacional, foram desenvolvidas curvas e recomendações nacionais adaptadas à diversidade da população brasileira. A nova ferramenta, de aplicação simples e vigente na rede pública desde 2022^12,14^, também pode ser utilizada em países com perfil semelhante^13^. Sua construção se baseou em dados de 21 estudos brasileiros do Consórcio *Brazilian Maternal and Child Nutrition Consortium* (BMCNC), com validação interna por meio do cruzamento com dados do Sistema de Vigilância Alimentar e Nutricional (SISVAN) e validação externa por meio do estudo “Nascer no Brasil”^12,15^.

Estudo com dados da coorte nacional “Nascer no Brasil”^16^ (n = 6.888) comparou as curvas brasileiras de GPG com três referências internacionais avaliando a predição de nascimentos PIG e GIG. As curvas de Hutcheon et al.^17^ foram desenvolvidas em amostra de mulheres norte-americanas; as do *Intergrowth-21st* ^18^, em mulheres saudáveis residentes em áreas urbanas de oito países (Brasil, China, Índia, Itália, Quênia, Omã, Reino Unido e Estados Unidos); e as do consórcio *Lifecycle* ^19^, com base em dados de 33 coortes da Europa, América do Norte e Oceania.

Em relação às referências avaliadas no estudo mencionado previamente, as curvas brasileiras apresentaram maior capacidade preditiva, com distribuição dos valores de GPG e proporções de mulheres classificadas abaixo e acima de percentis selecionados mais próximo do padrão esperado, considerando o IMC pré-gestacional e o trimestre gestacional. Em contraste, os gráficos do *Intergrowth-21st* ^18^ e *Lifecycle* ^19^ classificaram uma maior frequência de mulheres abaixo do percentil 50, enquanto os de Hutcheon et al.^17^ superestimaram o GPG em mulheres classificadas como eutróficas, sobrepeso e obesidade^16^.

Um ensaio clínico randomizado^20^ avaliou a efetividade de um aconselhamento nutricional na prevenção do GPG excessivo em gestantes com sobrepeso. A intervenção, baseada no incentivo ao consumo de alimentos *in natura* e minimamente processados em detrimento do consumo de ultraprocessados e a prática regular de atividade física, reduziu a chance de GPG excessivo (RC = 0,56; IC95% 0,32–0,98; p = 0,04). Contudo, o ganho ponderal materno não foi analisado conforme as recomendações nacionais vigentes.

Partindo do pressuposto de que as diretrizes da NAM, amplamente utilizadas no mundo, não foram validadas para a população brasileira e que podem subestimar o GPG excessivo, especialmente entre gestantes com sobrepeso, a hipótese deste estudo é que as curvas e recomendações brasileiras oferecem maior sensibilidade na identificação de padrões inadequados de GPG. A comparação entre os métodos é relevante, uma vez que gestantes com sobrepeso apresentam maior risco de ganho ponderal excessivo durante a gestação, condição associada a desfechos maternos e neonatais adversos^21^. Assim, o objetivo deste estudo foi comparar os métodos de avaliação do GPG propostos pelas diretrizes norte-americanas (NAM) e pelas curvas e recomendações brasileiras em gestantes com sobrepeso.

## MÉTODOS

### Delineamento do Estudo e População

Trata-se de uma análise secundária que empregou dados de um ensaio clínico randomizado controlado, realizado entre 2018 e 2021, com gestantes adultas com sobrepeso em acompanhamento pré-natal em sete unidades de saúde (US) do município de Ribeirão Preto, SP, Brasil. O protocolo do estudo encontra-se detalhadamente descrito na publicação de Sartorelli et al.^22^ A execução do ensaio clínico foi aprovada pelo Comitê de Ética em Pesquisa do Centro de Saúde Escola da Faculdade de Medicina de Ribeirão Preto (69997717.6.0000.5414) e seguiu as diretrizes do CONSORT. O ensaio clínico foi registrado no Registro Brasileiro de Ensaio Clínico em julho de 2018 (RBR-2w9bhc).

Nutricionistas capacitadas identificaram mulheres potencialmente elegíveis e as convidaram para participar da pesquisa no momento da consulta de pré-natal nas US. Foram incluídas gestantes de baixo risco com idade igual ou superior a 18 anos, IMC pré-gestacional entre 25 kg/m^2^ e 29 kg/m^2^ e idade gestacional de, no máximo, 15 semanas e 6 dias. Mulheres que relataram diabetes prévio ou uso atual de hipoglicemiantes orais ou insulina ou medicamento para perda de peso foram excluídas. Todas as participantes que concordaram em participar do estudo assinaram o Termo de Consentimento Livre e Esclarecido e foram aleatoriamente sorteadas no grupo de intervenção (GI) ou grupo controle (GC).

No ensaio clínico, a amostra foi calculada com base no desfecho primário proposto: a proporção de mulheres com GPG excessivo. Foram considerados um nível mínimo de significância de 5% (α = 0,05), um poder de 90% (β = 0,10) e uma perda de seguimento de 40% em decorrência do isolamento social imposto pela pandemia da covid-19, totalizando uma amostra de 350 gestantes.

No presente estudo, o tamanho amostral baseou-se na estratégia de amostragem não probabilística do tipo conveniente^23^, sendo incluídas todas as mulheres com dados para o cálculo do GPG cumulativo para classificação segundo as curvas brasileiras. Ademais, seguindo o princípio da intenção de tratar modificada, foram incluídas mulheres que participaram de pelo menos uma sessão de aconselhamento nutricional para aquelas do GI^24^. Assim, foram incluídas 260 gestantes (GI = 121; GC = 139) (Figura).

**Figura:** Fluxograma do estudo.

### Características Maternas

Dados sociodemográficos, obstétricos e de estilo de vida materno, como idade, estado civil, autorrelato da cor da pele, exercício de trabalho remunerado, paridade, prática semanal de atividades físicas, tabagismo e consumo de bebidas alcoólicas no último mês, foram coletados, por pesquisadoras nutricionistas treinadas, por meio de um questionário estruturado, durante a primeira avaliação do ensaio clínico.

A randomização das participantes foi realizada por meio do *software* REDCap^25^, com alocação estratificada por US, a fim de garantir distribuição equilibrada entre os grupos controle e intervenção. Os participantes e pesquisadores tinham ciência da alocação entre os grupos devido à natureza do aconselhamento nutricional, sessões presenciais com nutricionistas treinadas, que impossibilitava o cegamento.

Todas as participantes receberam atendimento pré-natal usual nas US, incluindo orientações sobre ganho de peso, alimentação e dúvidas gestacionais. O GI participou de três sessões individuais de aconselhamento nutricional, focadas no incentivo ao consumo de alimentos *in natura* e minimamente processados, em detrimento de ultraprocessados, e na prática regular de atividade física. As sessões, conduzidas por nutricionistas, duraram cerca de 30 minutos, com intervalos médios de seis semanas.

As gestantes do GC receberam apenas o pré-natal de rotina na US, que inclui avaliação do peso e da estatura, orientações gerais sobre alimentação saudável e ganho de peso adequado durante a gestação. Após o parto, ambos os grupos receberam orientação para recuperação do peso pré-gestacional. Detalhes adicionais estão descritos em publicações prévias^20,22^.

### Ganho de Peso Gestacional

O cálculo do GPG cumulativo foi feito a partir da diferença entre o último peso da participante, obtido em média às 33,5 semanas de gestação, e o peso pré-gestacional estimado. A última medida de peso da participante foi obtida na ocasião da segunda avaliação do ensaio clínico ou por meio do acesso ao prontuário médico no sistema de informação *HygiaWeb* (dado secundário). Foi realizado a correlação entre os valores de pesos mensurados na segunda avaliação do estudo e os valores secundários obtidos e obteve-se um coeficiente de correlação de *Spearman* de 0,99 (p < 0,01)^20^.

O peso pré-gestacional foi estimado com base no peso aferido no momento da triagem do ensaio clínico. O peso aferido até a 13ª semana gestacional (SG) foi adotado como peso pré-gestacional, se aferido entre a 14ª e 15ª SG, foram subtraídos, respectivamente, 0,45 kg e 0,91 kg do peso medido^26^. O IMC pré-gestacional foi calculado pela razão entre o peso pré-gestacional e a altura aferida ao quadrado, sendo classificado de acordo com os referenciais teóricos da Organização Mundial da Saúde, sendo o de 1995^27^ para gestantes acima de 19 anos, e para as gestantes adolescentes (n = 17) (≥ 18 anos e ≤ 19 anos) o referencial de classificação do IMC por idade^28^.

Para estimar o ganho de peso médio semanal, subtraiu-se o peso aferido na segunda avaliação do ensaio clínico, realizada preferencialmente entre a 34ª e a 36ª SG, do peso registrado durante o segundo trimestre da gestação (≥ 12ª SG). A diferença foi dividida pelo número de semanas entre os dois momentos. Na ausência de registro de peso entre a 34ª e a 36ª SG, utilizou-se a última aferição de peso disponível na consulta pré-natal anterior à 36ª SG, disponível no prontuário médico da gestante (dado secundário).

### Métodos de Avaliação para o Ganho de Peso Gestacional

#### Curvas e recomendações brasileiras

As curvas e recomendações de GPG para gestantes brasileiras foram empregadas para a avaliação do GPG materno^13^. A classificação do ganho ponderal cumulativo foi realizada por meio da determinação exata do percentil para um banco de dados de participantes, a partir das informações do ganho ponderal cumulativo, da idade gestacional na última medida de peso e do EN pré-gestacional. O percentil foi calculado utilizando o *software* Microsoft Excel^29^.

Devido a amostra ser composta exclusivamente por mulheres com sobrepeso pré-concepção, foi adotada a faixa de percentis de acordo com esse EN, em que gestantes com ganho de peso acima do percentil 27 foram classificadas com GPG excessivo, aquelas entre o percentil 18 e 27 com GPG adequado e as mulheres abaixo do percentil 18, classificadas com GPG insuficiente^30^.

#### National Academy of Medicine

De acordo com a proposta do NAM, o ganho de peso médio semanal foi classificado em: insuficiente, adequado e excessivo, considerando-se o intervalo de ganho ponderal recomendado para gestantes com sobrepeso pré-concepção (IMC entre 25 kg/m^2^ e 29,9 kg/m^2^). Dessa forma, gestantes com ganho de peso médio semanal inferior a 0,23 kg foram classificadas com GPG insuficiente, aquelas com GPG semanal superior a 0,33 kg com ganho de peso excessivo, e mulheres que apresentaram um ganho ponderal médio semanal entre 0,23 kg e 0,33 kg foram classificadas com GPG semanal adequado^2^.

## Análises Estatísticas

As características maternas referentes à linha de base do estudo foram apresentadas em frequência absoluta (n) e frequência relativa (%) e o teste qui-quadrado de Pearson foi empregado para verificar a associação destas variáveis, de acordo com os grupos de tratamento.

Para avaliar a concordância entre os dois métodos de classificação do GPG, NAM e as curvas de recomendações nacionais, foi utilizado o coeficiente de concordância Kappa ponderado^31^. O coeficiente de concordância de Kappa foi interpretado da seguinte maneira: menor que 0,20 representa concordância fraca; 0,20–0,40 representa concordância razoável; 0,41–0,60 representa concordância moderada; 0,61–0,80 representa concordância substancial; mais que 0,80 representa grande concordância^32^.

As análises deste estudo seguiram os princípios da intenção de tratar modificada^24^, nas quais as mulheres do GI que não participaram de qualquer sessão de aconselhamento nutricional foram excluídas. A abordagem foi adotada considerando que o GPG pode ser modificado por intervenções no pré-natal. Assim, para garantir a comparabilidade entre os grupos, o GI incluiu gestantes que participaram de ao menos uma sessão de aconselhamento nutricional, enquanto o GC não foi exposto à intervenção.

Modelos de regressão logística brutos foram empregados para comparar o GPG segundo os métodos de monitoramento e os grupos de tratamento, adotando-se a categoria de GPG adequado como referência. Para a seleção do conjunto de variáveis de ajustes, foi construído um *Directed Acyclic Graph* (DAG)^33^ incluindo a variável exposição (grupo de tratamento), desfecho (GPG) e outras variáveis maternas coletadas no estudo (Material Suplementar)^a^.

No presente estudo, o DAG indicou ausência de variáveis de confusão na associação entre grupo de tratamento e ganho ponderal gestacional, uma vez que se trata de um ensaio clínico controlado e randomizado, o que já minimiza tais vieses. Ajustes adicionais poderiam superestimar o controle e comprometer os resultados^34^. As análises foram realizadas no *software* SAS versão 9.4 para Windows (SAS Institute Inc., Cary, NC, USA) e valores de p < 0,05 foram considerados significativos.

## RESULTADOS

Das 335 gestantes com informações referentes à linha de base, considerando os grupos de tratamento (GI = 169 e GC = 166), 266 tinham dados de peso disponíveis para calcular o ganho ponderal cumulativo e semanal (GI = 127 e GC = 139). Entretanto, seguindo o princípio da intenção de tratar modificada, seis gestantes do GI foram excluídas das análises por não terem participado de nenhuma sessão de aconselhamento nutricional, totalizando em 121 gestantes no GI.

A maioria das gestantes tinha 25 anos ou menos. Em ambos os grupos, verificou-se maior frequência entre gestantes que praticavam menos de 150 minutos semanais de atividade física e se autodeclararam pardas. Observou-se diferença significativa entre os grupos quanto ao trabalho remunerado, com maior proporção de gestantes não exercendo atividade remunerada no GC. Para as demais características maternas, não foram observadas diferenças significativas entre os grupos (Tabela 1).

**Tabela 1:** Características maternas na linha de base de acordo com o grupo de tratamento. Ribeirão Preto (SP), 2021, n = 260.

Características maternas	GI (n = 121) n (%)	GC (n = 139) n (%)	p-valor^a^
Idade (anos)			0,55
≤ 25	47 (42,7)	63 (57,3)	
26–34	53 (48,6)	56 (51,4)	
≥ 35	21 (51,2)	20 (48,8)	
Prática de atividade física (minutos/semana)			0,78
< 150	86 (46,0)	101 (54,0)	
≥ 150	35 (48,0)	38 (52,0)	
Estado civil			0,42
Casada/mora com o companheiro	94 (48,0)	102 (52,0)	
Outras	27 (42,2)	37 (57,8)	
Autorrelato cor da pele			0,81
Branca	36 (44,4)	45 (55,6)	
Preta	17 (45,9)	20 (54,1)	
Parda	66 (47,8)	72 (52,2)	
Escolaridade (anos de estudo)^b^			0,94
≤ 8	28 (48,3)	30 (51,7)	
9 a 11	79 (45,9)	93 (54,1)	
≥ 12	14 (48,3)	15 (51,7)	
Trabalho remunerado			**0,02**
Sim	83 (52,5)	75 (47,5)	
Não	38 (37,3)	64 (62,7)	
Paridade			0,19
Nulíparas	40 (41,2)	57 (58,8)	
Multípara	81 (49,7)	82 (50,3)	
Tabagismo			0,41
Nunca fumou	93 (48,9)	97 (51,1)	
Ex-fumante	10 (37,0)	17 (63,0)	
Fuma atualmente	18 (41,9)	25 (58,1)	
Consumo de álcool nos últimos 30 dias			0,57
Sim	21 (42,9)	28 (57,1)	
Não	100 (47,4)	111 (52,6)	

GI: grupo intervenção; GC: grupo controle.

^a^ Referente ao teste qui-quadrado de Pearson.

^b^ Dados disponíveis para 259 mulheres.

Os métodos de monitoramento do GPG, segundo os grupos de tratamento, apresentaram concordância moderada, conforme indicado pelos coeficientes de concordância kappa. Entre as 139 mulheres do GC, 116 (83,45%) foram classificadas por ambos os métodos na mesma categoria de GPG. Em relação ao GI, das 121 mulheres, 97 (80,17%) foram classificadas na mesma categoria de GPG pelos dois métodos (Tabela 2).

**Tabela 2:** Análise de concordância entre os métodos de monitoramento do ganho de peso gestacional segundo os grupos de tratamento. Ribeirão Preto (SP), 2021, n = 260.

Curvas brasileiras (2021)	NAM (2009)			Kappa
	Grupo controle (n = 139)			
	Insuficiente (n = 12)	Adequado (n = 25)	Excessivo (n = 102)	
Insuficiente (n = 12)	**10 (83,3)**	2 (8,0)	0 (0)	
Adequado (n = 12)	2 (16,7)	**7 (28,0)**	3 (2,9)	0,55 (IC95% 0,40–0,70)
Excessivo (n = 115)	0 (0)	16 (64,0)	**99 (97,1)**	
	Grupo intervenção (n = 121)			
	Insuficiente (n = 17)	Adequado (n = 29)	Excessivo (n = 75)	
Insuficiente (n = 20)	**16 (94,1)**	4 (13,8)	0 (0)	
Adequado (n = 7)	1 (5,9)	**6 (20,7)**	0 (0)	0,58 (IC95% 0,46–0,72)
Excessivo (n = 94)	0 (0)	19 (65,5)	**75 (100)**	

NAM: *National Academy of Medicine*; IC95%: intervalo de confiança de 95%.

Em negrito: frequências concordantes entre os métodos.

Fontes: Kac et al.^13^ (2021); IOM (2009)^2^.

Com pontos situados abaixo da diagonal principal (em negrito), identificou-se um possível padrão de subestimação do GPG pelo método norte-americano, independente do grupo de tratamento. Nesse sentido, no GC duas gestantes (16,7%) foram classificadas com ganho de peso adequado pelo método das curvas brasileiras e já pelo método NAM, com ganho de peso insuficiente. E ainda, 16 gestantes (64,0%) com ganho de peso excessivo pelas curvas brasileiras, foram classificadas com ganho de peso adequado pelo NAM. Enquanto no GI, uma mulher (5,9%) foi categorizada com GPG adequado pelo método nacional e insuficiente pelo método NAM e 19 mulheres (65,5%) com GPG excessivo pelo método nacional, foram categorizadas como GPG adequado pelo método norte-americano (Tabela 2).

A Tabela 3 apresenta os dados de avaliação do GPG segundo os métodos de monitoramento, considerando os grupos de tratamento. Observou-se que de acordo com o método das curvas brasileiras as mulheres do GI, em comparação ao GC, apresentaram maior chance de GPG insuficiente (RC = 2,87; IC95% 0,88–9,25) e segundo o método NAM menor chance de GPG excessivo (RC = 0,63; IC95% 0,34–1,17), indicando associações em direções opostas e diferentes magnitudes, embora sem significância estatística nos dois métodos.

**Tabela 3:** Avaliação do ganho de peso gestacional segundo os métodos de monitoramento de ganho de peso gestacional por grupos de tratamento. Ribeirão Preto (SP), 2021, n = 260.

Métodos	GC	GI	p-valor^a^	RC^b^ (IC95%)
NAM	GPG n (%)			
Insuficiente	12 (8,6)	17 (14,0)	0,13	1,22 (0,49–3,04)
Adequado	25 (18,0)	29 (24,0)		1,00 (ref.)
Excessivo	102 (73,4)	75 (62,0)		0,63 (0,34–1,17)
Curvas brasileiras				
Insuficiente	12 (8,6)	20 (16,5)	0,12	2,87 (0,88 –9,25)
Adequado	12 (8,6)	7 (5,8)		1,00 (ref.)
Excessivo	115 (82,7)	94 (77,7)		1,40 (0,53 –3,70)

GPG: ganho de peso gestacional; IC95%: intervalo de confiança de 95%; GC: grupo controle; GI: grupo intervenção; NAM: *National Academy of Medicine*; RC: razão de chances; ref.: referência.

^a^Teste qui-quadrado de Pearson.

^b^ Razão de chances calculado por modelos de regressão logística, GI grupo de referência (efeito da intervenção comparado ao controle).

## DISCUSSÃO

Este foi o primeiro estudo nacional a comparar o método de monitoramento do GPG pelas curvas brasileiras com o método proposto pela NAM entre gestantes com sobrepeso. Observou-se uma concordância moderada entre os métodos pelo coeficiente de concordância kappa, assim como uma tendência de subestimação do GPG pelo método norte-americano. Além disso, pelo método brasileiro, observou-se um aumento na chance de GPG insuficiente entre as gestantes do GI, enquanto o método norte-americano indicou menor chance de ganho excessivo nesse mesmo grupo, embora ambos sem significância estatística. Esses achados podem reforçar o padrão de subestimação associado ao método norte-americano em comparação às curvas brasileiras, uma vez que o método classificou um número menor de gestantes com ganho ponderal excessivo no grupo intervenção.

Em relação à concordância moderada observada entre os métodos, não há na literatura estudos que tenham explorado essa investigação. No entanto, há pesquisas que analisaram a capacidade preditiva desses métodos quanto aos desfechos de saúde maternos e fetais^7,16,35,36^ e indicam desempenho preditivo limitado ou modesto, especialmente quando utilizados de forma isolada. Além disso, sugerem que gráficos locais ou regionais tendem a representar melhor o padrão da população avaliada, o que pode conferir maior poder preditivo em comparação a recomendações internacionais.

Evidências têm destacado que as recomendações de GPG total e ganho médio semanal propostas pela NAM podem não ser ideais para gestantes não norte-americanas, principalmente em países não desenvolvidos^2,36^. Uma revisão de estudos na América Latina comparou a precisão de três propostas de avaliação do ganho ponderal materno na identificação de risco para inadequações de comprimento e peso ao nascer: (1) o *Institute of Medicine* (IOM) – atual NAM, (2) gráfico de Rosso-Mardones (RM) e (3) gráfico de RM modificado, proposto por Atalah et al.^36^

Em grandes amostras de gestantes chilenas e uruguaias, identificaram maior sensibilidade do gráfico RM para identificar casos de risco. Contudo, valores preditivos foram similares para as três propostas, em geral, uma baixa capacidade preditiva positiva e uma alta capacidade preditiva negativa para os desfechos analisados^36^. Segundo os autores, a principal limitação para o uso das recomendações de ganho ponderal do IOM em mulheres latino-americanas está relacionada à menor estatura média dessa população, aproximadamente 20 cm inferior à observada entre mulheres norte-americanas, variável que compõe o cálculo do IMC^5^ e que também pode influenciar diretamente o peso ao nascer^39^. De forma semelhante, a altura média das mulheres brasileiras é bem inferior à de mulheres norte-americanas (160 cm *versus* 176 cm, respectivamente)^5,40^.

Nesse sentido, os achados do presente estudo são consistentes com a literatura prévia, ao evidenciar concordância apenas moderada entre os métodos e uma tendência de subestimação do GPG pelo método norte-americano em comparação às curvas brasileiras. Essa tendência pode levar à classificação de gestantes brasileiras como apresentando ganho insuficiente quando o ganho observado é compatível com o padrão antropométrico e biossocial nacional, além de reduzir a identificação de ganho ponderal excessivo, particularmente no GI.

Em uma coorte retrospectiva realizada na China^35^, com 13.366 gestantes, diferentes padrões de GPG (*Intergrowth-21st*, IOM e uma referência chinesa) foram comparados quanto à capacidade de identificar o risco de DMG e de desfechos adversos relacionados. Entre as gestantes, 46,0% ganharam peso acima das recomendações do IOM, enquanto 40,0% e 30,0% ganharam peso acima das recomendações do *Intergrowth-21st* e da referência chinesa, respectivamente. Por outro lado, foi observado que gestantes com ganho ponderal acima das recomendações para os padrões *Integrowth-21st* e da referência chinesa apresentaram riscos aumentados de DMG, de 27,0% e 30,0%, respectivamente. Enquanto gestantes com GPG excessivo segundo as recomendações do IOM apresentaram risco aumentado de 22,0% de DMG, representando um menor poder de predição.

Ademais, a literatura discute se as recomendações de ganho ponderal da NAM são adequadas para todas as classes de IMC. Na Alemanha, um estudo com mais de 650.000 partos avaliou sua validade, comparando a prevalência de parto prematuro e baixo peso ao nascer entre mulheres que ganharam peso dentro ou fora das recomendações, estratificadas por IMC pré-gestacional. Mulheres com baixo peso e eutróficas que seguiram as recomendações apresentaram menor prevalência de desfechos adversos. Já entre mulheres com sobrepeso e obesidade, seguir as diretrizes associou-se a maior risco. Concluiu-se que a validade das recomendações varia conforme o IMC pré-concepção, ressaltando a necessidade de diretrizes específicas para cada grupo^38^.

Tsai et al.^41^ avaliaram a adequação das recomendações da NAM para mulheres taiwanesas, analisando o risco de macrossomia fetal. Mulheres eutróficas que ganharam > 13 kg apresentaram risco quatro vezes maior de macrossomia, e mulheres com sobrepeso que ganharam > 11,5 kg tiveram risco nove vezes maior. Os autores sugeriram limites mais baixos: < 11,5 kg para eutróficas e < 10 kg para mulheres com sobrepeso, sendo estes valores inferiores aos da NAM, que podem subestimar o ganho excessivo e aumentar o risco de desfechos neonatais adversos.

No presente estudo, embora não tenha sido encontrada nenhuma diferença significativa no GPG entre os grupos de tratamento, independentemente do método empregado, observou-se um aumento na chance de GPG insuficiente entre as gestantes do GI pelo método brasileiro, enquanto o método norte-americano indicou menor chance de ganho excessivo nesse mesmo grupo.

Em uma revisão de ensaios clínicos randomizados envolvendo 11 estudos (n = 4.422 gestantes), verificou-se que intervenções baseadas em dieta, exercícios ou ambos aumentaram o risco de GPG insuficiente em comparação ao controle (RR = 1,14; IC95% 1,02–1,27)^42^. Entretanto, no presente estudo, o GPG insuficiente pode ter sido superestimado em função da metodologia utilizada para estimar o peso pré-gestacional, obtido por aferição até a 13ª semana de gestação, sem considerar o ganho ponderal inicial^26^. Evidências recentes indicam que o peso aferido até a 8ª semana ou o peso autorreferido podem fornecer estimativas mais precisas do peso pré-gestacional^15^. Outra explicação plausível é o fato de que algumas mulheres do GI possam ter apresentado uma maior preocupação diante do aconselhamento nutricional, em virtude do bem-estar materno-infantil^43^.

O possível padrão de subestimação do GPG pelo método NAM pode ter contribuído para a menor proporção de gestantes classificadas com ganho excessivo e, consequentemente, para a observação de um efeito protetor da intervenção, ainda que sem significância estatística; uma vez que o GPG excessivo foi desfecho primário no estudo de intervenção. Isso pode ser justificado pelos limiares mais permissivos estabelecidos pela NAM em comparação às recomendações brasileiras, que são mais críticos e classificaram uma maior proporção de gestantes com ganho excessivo.

Nesse contexto, pequenas reduções no ganho ponderal materno, resultantes da intervenção, podem ter sido suficientes para reduzir o número de casos classificados como ganho excessivo segundo os critérios da NAM. No entanto, essas reduções não foram suficientemente expressivas para impactar a maior proporção de mulheres classificadas com ganho excessivo segundo as curvas brasileiras. Dessa forma, a divergência entre os métodos pode refletir diferenças na categorização do ganho ponderal gestacional, sem necessariamente indicar variações no efeito da intervenção sobre esse desfecho.

No estudo original^20^, a intervenção mostrou-se efetiva em reduzir a chance de GPG excessivo, avaliado segundo a NAM. No presente estudo essa associação não alcançou significância estatística. Isso pode estar relacionado ao fato de que, nas análises prévias, o DAG não foi empregado, havendo ajustes nos modelos. No presente estudo, o uso do DAG (material suplementar) evidenciou a ausência de variáveis de ajuste, uma vez que se trata de um ensaio clínico controlado e randomizado, o que por si só contribui para a minimização de vieses.

Nossos achados evidenciam uma divergência entre os métodos de monitoramento do GPG, não apenas quanto à concordância, mas também na avaliação do GPG entre os grupos de tratamento. Apesar da concordância moderada, verificou-se um padrão de classificação abaixo da diagonal principal, indicando que o método norte-americano tende a subestimar o ganho de peso excessivo em comparação às curvas brasileiras. Essa discordância se reflete em associações em direções opostas e de magnitude modesta, uma vez que o método brasileiro identificou maior chance de GPG insuficiente no GI (RC = 2,87), enquanto o método NAM apontou menor chance de GPG excessivo (RC = 0,63) nesse mesmo grupo.

Embora sem significância estatística, essa diferença é clinicamente relevante, uma vez que o método adotado para o monitoramento pode influenciar a identificação de gestantes em risco, ou seja, aquelas com ganho de peso acima do recomendado, impedindo intervenções nutricionais oportunas e um monitoramento mais rigoroso, capazes de prevenir desfechos adversos maternos e fetais. Adicionalmente, essas diferenças podem impactar a vigilância nutricional e comprometer políticas públicas voltadas à prevenção do ganho de peso excessivo e de suas consequências para a saúde.

O estudo apresenta algumas limitações: foi realizado exclusivamente com gestantes com sobrepeso, não podendo ser generalizado para mulheres em outras faixas de IMC pré-gestacional; a metodologia empregada na estimativa do peso pré-gestacional pode ter implicado uma superestimação do GPG insuficiente; o tamanho amostral deriva de um ensaio clínico não originalmente planejado para este desfecho, embora inclua todas as participantes com dados disponíveis e utilize um dos métodos considerado no cálculo amostral original (NAM); o cegamento não foi possível devido à natureza presencial da intervenção; e foi adotada intenção de tratar modificada para garantir a comparabilidade entre os grupos.

Dentro os pontos fortes do estudo, destacam-se a comparação entre o método nacional de monitoramento do GPG, recentemente incorporado no acompanhamento de gestantes na rede pública do país, frente ao método norte-americano anteriormente empregado. Além disso, a comparação de métodos em uma população de gestantes com sobrepeso é fundamental no âmbito da saúde pública, pois compreendem o grupo de maior risco de ganho ponderal excessivo na gestação^44,45^.

As análises de concordância indicaram que o método norte-americano tende a subestimar o GPG em comparação ao método brasileiro. Esse resultado evidencia limitações na aplicabilidade das diretrizes da NAM a populações distintas da norte-americana. Ademais, a ausência de validação dessas diretrizes para a população brasileira pode contribuir para uma classificação inadequada do GPG, sobretudo nos casos de ganho inadequado (insuficiente ou excessivo).

Portanto, as curvas e recomendações brasileiras representam um método de monitoramento mais fidedigno e adequado ao perfil das gestantes brasileiras. Sua ampla divulgação entre os profissionais de saúde é imprescindível para garantir um acompanhamento e evolução adequados na gestação, promovendo o bem-estar e a saúde do binômio mãe e filho.

## Material suplementar

Material suplementar

## Data Availability

Os dados que fundamentam os resultados apresentados neste estudo não estão disponíveis publicamente, mas podem ser disponibilizados mediante solicitação à autora correspondente.

## Footnotes

a Material Suplementar está disponível no Figshare: [DOI] 10.6084/m9.figshare.31851868 LINK: https://figshare.com/s/c2f4b96ca5432eb88ee5
